# Large Stokes Shift and High Efficiency Luminescent Solar Concentrator Incorporated with CuInS_2_/ZnS Quantum Dots

**DOI:** 10.1038/srep17777

**Published:** 2015-12-08

**Authors:** Chen Li, Wei Chen, Dan Wu, Dunhang Quan, Ziming Zhou, Junjie Hao, Jing Qin, Yiwen Li, Zhubing He, Kai Wang

**Affiliations:** 1 Department of Electrical and Electronic Engineering, South University of Science and Technology of China, Shenzhen, 518055, China; 2 School of Electrical and Electronic Engineering, Nanyang Technological University, Singapore, 639798, Singapore; 3 Department of Materials Science and Engineering, South University of Science and Technology of China, Shenzhen, 518055, China; 4 Shenzhen Key Laboratory of 3rd Generation Semiconductor Devices (SUSTC), Shenzhen, 518055, China

## Abstract

Luminescent solar concentrator (LSC) incorporated with quantum dots (QDs) have been widely regarded as one of the most important development trends of cost-effective solar energy. In this study, for the first time we report a new QDs-LSC integrated with heavy metal free CuInS_2_/ZnS core/shell QDs with large Stokes shift and high optical efficiency. The as-prepared CuInS_2_/ZnS QDs possess advantages of high photoluminescence quantum yield of 81% and large Stocks shift more than 150 nm. The optical efficiency of CuInS_2_/ZnS QDs-LSC reaches as high as 26.5%. Moreover, the power conversion efficiency of the QDs-LSC-PV device reaches more than 3 folds to that of pure PMMA-PV device. Furthermore, the PV device is able to harvest 4.91 folds solar energy with the assistance of this new CuInS_2_/ZnS QDs-LSC for the same size c-Si PV cell. The results demonstrate that this new CuInS_2_/ZnS QDs-LSC provides a promising way for the high efficiency, nonhazardous and low cost solar energy.

Lowering the cost of generating per unit power is one of the most important issues in global solar photovoltaic (PV) energy technology today in which fundamental researches toward reaching high conversion efficiency goes hand in hand with those on lowering production cost^1^,^2^,^3^. Concentrating sunlight is considered as an important way to decrease the cost of PV electricity generation, since the rise of the energy density will reduce the usage area of PV cells dramatically. Furthermore, conversion of the incident solar spectrum to material related absorption wavelength ranges which have efficiently PV effect would greatly increase the efficiency of PV cells. Therefore, the concept of luminescent solar concentrator (LSC) was proposed in solar energy conversion to serve the above beneficial purposes.

LSC was initially suggested in the late 1970 s which had potential to enhance the economic viability of solar energy^4^,^5^,^6^,^7^. Basically it consists of a highly transparent planar sheet incorporated with suitable luminophores, which including fluorescent organic dye molecules or inorganic quantum dots (QDs) that absorb the incident sunlight and re-emit it at longer wavelength which could be absorbed by PV cells more efficiently. LSC could concentrate both direct and diffuse incident sunlight and then guide towards the side edges through the total internal reflection (TIR). This design is attractive when it is implemented with high efficiency compound semiconductor PV cells at the edges of LSC, which named LSC-PV device. LSC-PV device could reduce the cost of solar energy by not only allowing replacement of expensive large area PV cells with cheaper solar-harvesting antennae coupled to the small ones, but also reaching high conversion efficiency. What’s more, it can convert high energy photons, which less absorbed by PV cells, into low energy photons, which more absorbed by PV cells, by the luminophores and further improve the conversion efficiency of PV cells. Figure 1(a) shows schematic of QDs-LSC edge-attached with PV cell and illustrates the principle of the device. This system can be integrated into the electronic displays, solar windows as well as other glazing systems^8^. Therefore, it will be a cost-effective alternative to optics-based solar concentration systems. Figure 1(b) illustrates three major behaviors of light in the QDs-LSC device, including light converting by QDs, Rayleigh scattering by nanoscale particles (QDs) and light guiding due to the TIR.

In recent years, many efforts have been made to increase the efficiency of LSCs, especially focusing on many promising luminophores for applications in LSCs. Liu *et al*. demonstrated that multiple organic dyes doped into LSC would enhance the absorption and efficiency^9^. Tummeltshammer *et al*. reported a new way to brighten LSC through homeotropic alignment and Förster resonance energy transfer between organic dyes and liquid crystals^10^. Organic dyes doped LSC was intensively investigated until researches highlighted the limited properties of organic dyes, such as narrow absorption band, poor photo-stability and large reabsorption. QDs doped LSCs have advantages over organic dyes since QDs possess a larger absorption region than that of dyes and are able to tune the absorption and emission spectra simply by adjusting core diameter and, being crystalline semiconductors, they are more stable and less degradable than organic dyes. Meinardi *et al*. introduced ‘Stokes-shift-engineered’ core/shell CdSe/CdS QDs which allow absorption and emission tunable across the entire solar spectra for incorporation in LSC^11^. Wilton *et al*. reported using PbSe QDs as the active fluorescent material and the self-absorption could be reduced by utilizing Förster resonance energy transfer between two different sizes of PbSe QDs^12^. Bradshaw *et al*. proposed to minimize reabsorption in large scale LSC by the doped Cd_1-*x*_Cu_*x*_Se QDs^13^. However, the wide employment of heave metal such as Cd and Pb in QDs-LSC devices would be harmful to the environment as well as to our human beings.

It is true that different applications demand specifically tailored luminophores, the achievements of commercial viability in various LSC configurations require new luminophores that possess high efficiency, low reabsorption and nontoxicity. CuInS_2_/ZnS core/shell QDs possess high optical absorption coefficient facilitating extensive utilization of the solar spectrum, large Stokes shift, broadband luminescence, superior stability under solar radiation and direct band gap of 1.5 eV which overlaps well with solar spectrum as well as absence of toxic elements^14^,^15^,^16^,^17^. In this study, we designed to assess the viability of heavy metal ions free CuInS_2_/ZnS core/shell QDs as the luminophores for LSC device for the first time to the best of our knowledge. A new LSC integrated with CuInS_2_/ZnS core/shell QDs with large Stokes shift and high optical efficiency was proposed. Moreover, the power conversion efficiency (PCE) of the PV cell attached at the side of the LSC at simulated sunlight also increased significantly comparing to that of PV cell with pure polymethyl methacrylate (PMMA) as light guider.

## Results and Discussions

**Figure 2:**
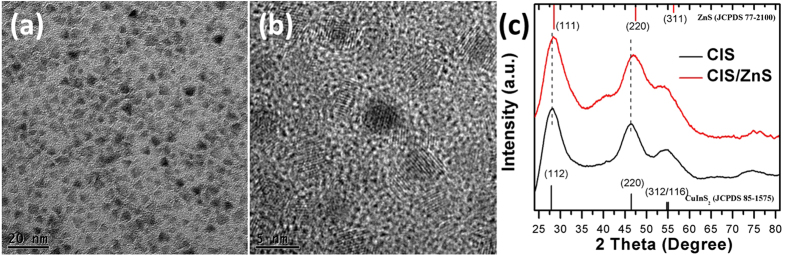
(**a**) TEM and (**b**) HR-TEM images of the as-synthesized CuInS_2_/ZnS core/shell QDs. (**c**) XRD pattern of CuInS_2_ and CuInS_2_/ZnS QDs.

Colloidal CuInS_2_/ZnS core/shell QDs were synthesized via one-pot method. By using the air-stable non-coordinating solvent paraffin liquid to slow down the reaction rate, a spherical shape and nearly monodispersed CuInS_2_/ZnS QDs with the average size of 4.0 ± 0.2 nm were obtained as shown in the Transmission Electron Microscopy (TEM) image, Fig. 2(a) and High Resolution TEM (HRTEM) image, Fig. 2(b). The X-Ray Diffraction (XRD) pattern of CuInS_2_ and CuInS_2_/ZnS QDs are illustrated in Fig. 2(c). The main peaks of CuInS_2_ (CIS) are observed to move towards larger angel for CuInS_2_/ZnS (CIS/ZnS) indicating the well inorganic alloyed and coating^18^,^19^.

**Figure 3:**
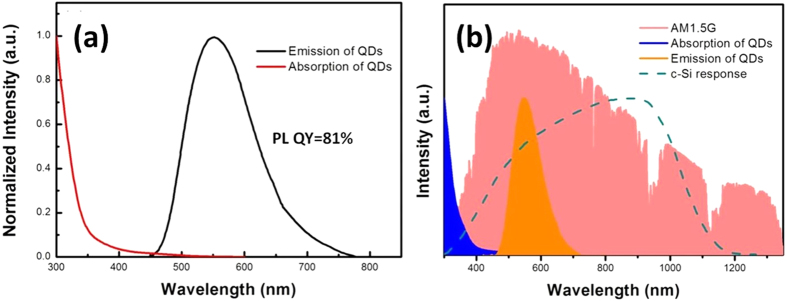
(**a**) Normalized absorption and emission spectra of CuInS_2_/ZnS QDs. (**b**) The relationships of AM 1.5 G spectrum, c-Si PV cells responsive spectrum and the absorption as well as emission spectra of CuInS_2_/ZnS QDs.

**Figure 4:**
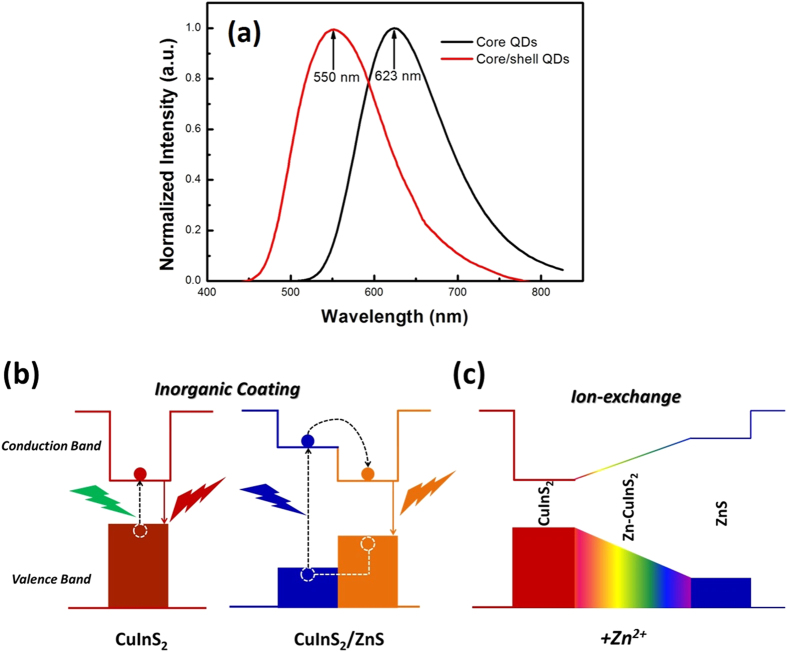
(**a**) Normalized emission spectra of CuInS_2_ core QDs and CuInS_2_/ZnS core/shell QDs. (**b**) Photoluminescence emission mechanism of CuInS_2_/ZnS core/shell QDs. (**c**) Schematic of the changes of energy gap with the addition of Zn^2+^.

Figure 3(a) shows the normalized absorption and emission spectra of CuInS_2_/ZnS QDs in chloroform solution. The emitting peak of CuInS_2_/ZnS QDs is observed at 550 nm and the full width at half maximum (FWHM) is about 125 nm. Moreover, the photoluminescence quantum yield (PL QY) of the QDs reaches to 81%. More importantly, combining with the PL spectrum and absorption spectrum, CuInS_2_/ZnS QDs are considered to possess weak self-absorption due to the core-shell structure of QDs^20^,^21^ and the Stocks shift reaches as large as more than 150 nm. We can find from Fig. 4(b) that, for the CuInS_2_/ZnS QDs, the emission wavelength is mainly dominated by the CuInS_2_ core and Zn-CuInS_2_ nanocrystals, while the absorption wavelength is mainly dominated by the ZnS shell with wider band gap since it has much larger amount than that of core material. In addition, Fig. 3(b) demonstrates that CuInS_2_/ZnS QDs are able to convert light with wavelength less than 450 nm into light with wavelength around 550 nm effectively, which could be absorbed by the c-Si PV cells more efficiently.

The emission wavelength was observed to blue shift from 623 nm for CuInS_2_ QDs to 550 nm for CuInS_2_/ZnS QDs as shown in Fig. 4(a), while the PL QY value was consequently improved from 23% for pure CuInS_2_ QDs to 81% for CuInS_2_/ZnS QDs with ZnS shell coating. This phenomenon is partially different from the typical binary QDs, such as CdSe for inorganic coating^22^,^23^,^24^, especially for the significant blue shift of emission wavelength after ZnS shell coating. Typical binary QDs’ inorganic coating mechanism is shown in Fig. 4(b). Wider band gap materials, such as ZnS, are always acting as shell by epitaxial growth on cores with lower valence band and higher conduction band than that of core materials to obtain the core-shell structures. The exciton will be well confined in the core’s band gap as shown in Fig. 4(b) and the coated QDs is no longer sensitive to longer wavelength photos due to the interaction of outer shell absorption character in this core-shell structure. Moreover, with the inorganic coating, the surface defects states, such as dangling bands, surface imperfections, etc., will be removed efficiently which is beneficial to the exciton radiate recombination and resulting in the increase of PL QY value of QDs. For CuInS_2_/ZnS QDs, besides the benefits of inorganic shell coating for typical binary QDs such as low self-absorption and high PL QY, the ion exchange effect is implemented to explain the blue shift phenomenon showing in Fig. 4(c). The Zn^2+^ ions are used to form the ZnS inorganic shell and incorporating into to CuInS_2_ lattice structure to replace the Cu^+^ and In^3+^ and therefore reduce the core region resulting in the blue shift since the quantum size effect. Meanwhile, the gradual Zn^2+^ incorporating brings alloying effect by the formation of Zn-CuInS_2_ nanocrystals which possess a broader emission band as shown in Fig. 4(c). Increasing amount of Zn^2+^ incorporating into CuInS_2_ results in emission band enlargement and emission peak blue-shift for the final CuInS_2_/ZnS QDs^18^,^25^,^26^.

**Figure 5:**
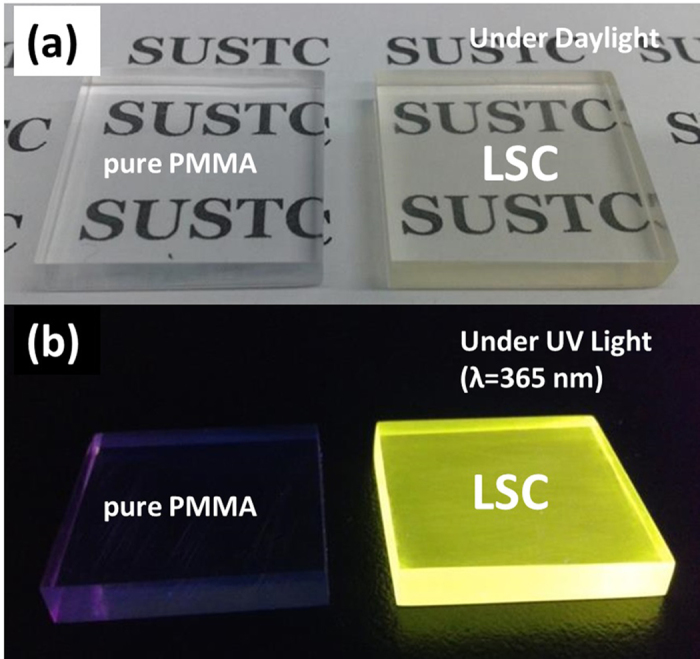
Photographs of the LSC prototype (22 mm × 22 mm × 3 mm) non-containing and containing CuInS_2_/ZnS QDs (a) under daylight and (b) illuminated by UV lamp.

In order to evaluate the performance of these QDs in the application of LSC device, we have fabricated a CuInS_2_/ZnS QDs-LSC prototype sized as 22 mm × 22 mm × 3 mm, yielding a geometric gain (denoted as G, the surface area of the top face divided by the surface area of the edges) of 1.83, by incorporating the CuInS_2_/ZnS QDs into the PMMA matrix by an *in-situ* polymerization method described in the experimental section. Figure 5 presents the photographs of the CuInS_2_/ZnS QDs-LSC devices under daylight and ultraviolet (UV) light, where the left sample is pure PMMA plate and the right sample is the LSC with QDs’ incorporation. The LSC reveals good transparency under daylight and luminescence performance under UV light.

**Figure 6:**
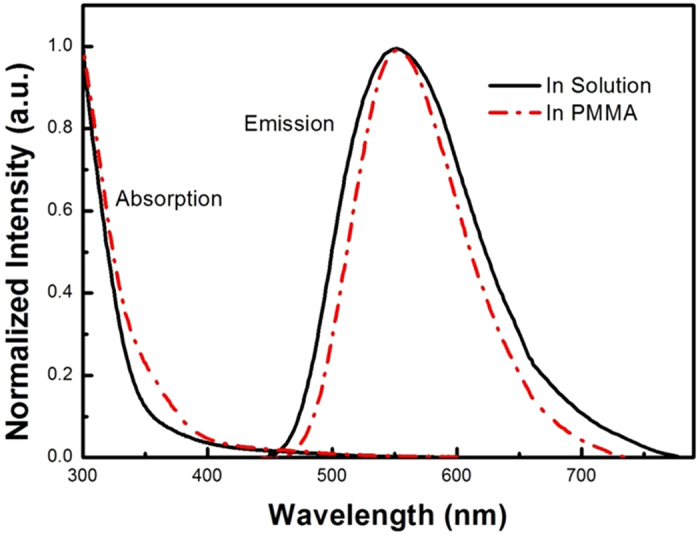
Normalized absorption and emission spectra of the CuInS_2_/ZnS QDs in solution (solid line) and PMMA composite (dot dash line).

Figure 6 presents the absorption and emission spectra of the CuInS_2_/ZnS QDs in solution and PMMA matrix. The QDs-LSC has successfully maintained the emission peak of original QDs. In addition, the decease of FWHM (about 8 nm) for QDs from solution to solid was observed due to the circumstance alteration. As known from literature, CuInS_2_ QDs intrinsically possess broad emission due to not only the size distribution but also the distinct lattice vibration^27^. We assume that the vibration behavior would be more restricted in solid PMMA matrix than that in solution resulting in the narrower FWHM for QDs-LSC’s PL emission to that of QDs in liquid.

Moreover, the PL QY of QDs-PMMA composite was measured as 56% which was somewhat lower than that of QDs in solution possibly due to the alterations of QDs’ concentration from dilute to dense and circumstance from liquid to solid. QDs’ luminescence properties had successfully maintained to a large extant during the chemical polymerization. The good performances of CuInS_2_/ZnS QDs in PMMA demonstrate they have very potential to be applied in LSC device.

The behavior of a QDs-LSC device under different excitation wavelength is usually to be evaluated in terms of its optical efficiency *η*, which defines as the number of photons emitted from sides of QDs-LSC device (*N*_*em*_) divided by the total number of photons absorbed by the QDs-LSC device (*N*_*ab*_) as Equation 1 as follows:


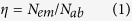


An integrating sphere system is adopted to measure the spectrum as well as the optical power of the light emitted from and absorbed by the QDs-LSC device so as to obtain *η*. The calculation of *η* is defined as Equations 2 and 3 as follows:


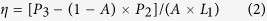



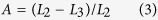


where *L*_*1*_ is the number of excitation photons while no sample is in the integrating sphere, and *L*_*2*_ and *P*_*2*_ are the number of excitation photons that not absorbed and the number of emission photons that emitted from the sample while it is not directly illuminated by the excitation light, respectively. Similarly, *L*_*3*_ and *P*_*3*_ are the number of excitation photons that not absorbed and the number of emission photons that emitted from the sample while it is directly illuminated by the excitation light, respectively.

**Figure 7:**
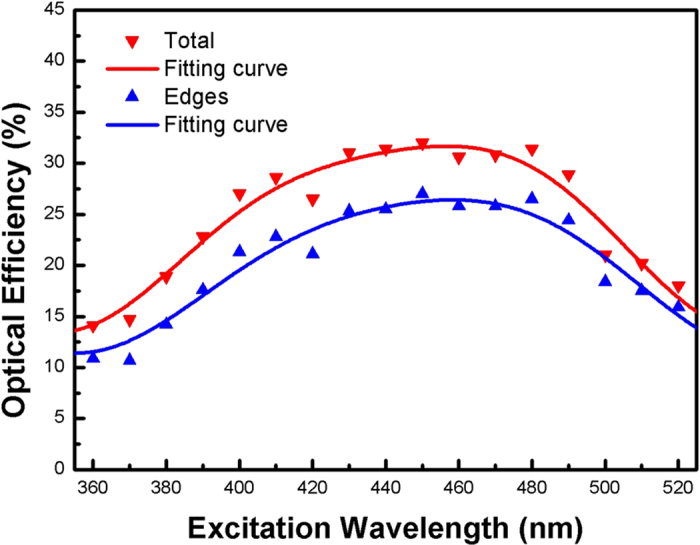
Optical efficiency of the QDs-LSC prototype against different excitation wavelength. Red triangle and line (fitting curve) are the total optical efficiency *η*_1_, and blue triangle and line (fitting curve) are the optical efficiency *η* considering edges only.

The values of *η* as a function of excitation wavelengths are shown in Fig. 7, which have been measured using an integrating sphere with diameter of 120 mm. The process of measurement can be summarized by two steps. Firstly, the total optical efficiency from all surfaces of CuInS_2_/ZnS QDs-LSC device was measured, defined as *η*_1_. Secondly, black carbon paint was used to cover the edges of the device such that the light could only be emitted from the top and bottom faces of the device, and the optical efficiency *η*_2_ was then measured again as previously stated. By subtracting *η*_2_ from *η*_1_, the final optical efficiency *η* (*η* = *η*_1_ − *η*_2_) was obtained, defined here as the fraction of the emission from the edges alone.

As we can see from Fig. 7, the value of *η* is dependent on the excitation wavelengths and the maximum value of *η* locates at 460 nm (fitting curves) reaching as high as 26.5%. These are comprehensive results affected by several factors simultaneously, including different responses of CuInS_2_/ZnS QDs excited by excitation light with different wavelengths, different absorption of the PMMA matrix of LSC as well as different light scattering behavior of nanoparticles (e.g. QDs) against different wavelengths. Moreover, the maximum response point is near 460 nm which indicates that the QDs-LSC device will be most efficient for light converting and light guiding near the specified wavelength from the sunlight.

**Figure 8:**
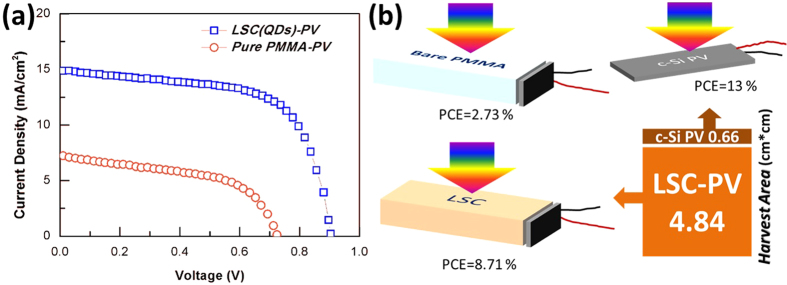
(**a**) J–V characteristics of c-Si PV cells combined with pure PMMA as well as CuInS_2_/ZnS QDs-LSC with the same size of 22 mm × 22 mm ×3 mm for light harvest under AM 1.5 G illuminated. (**b**) Different schematic of LSC-PV devices.

In order to well perform the new CuInS_2_/ZnS QDs-LSC, we assembled two LSC-PV devices, QDs-LSC-PV and pure PMMA-PV, as shown in Fig. 8(b). The CuInS_2_/ZnS QDs-LSC and the pure-PMMA plate possess the same size and are integrated with the same commercial c-Si PV cell with the given PCE of 13%. These devices were illuminated by a solar simulator with an air mass 1.5 global illumination (AM 1.5 G, 100 mW/cm^2^) during experiments. Photocurrent density-voltage (J–V) curve is a very important characterization to evaluate the performance of one PV device. The J–V curves as well as other important parameters, including open-circuit voltage (V_oc_), short-circuit current density (J_sc_), fill factor (FF) and PCE, of these two different LSC-PV devices are provided in Fig. 8(a) and Table 1. We can find that the photocurrent density increases significantly as the incorporation of CuInS_2_/ZnS QDs. In details, the values of V_oc_ and J_sc_ increase from 0.72 V and 7.2 mA/cm^2^ to 0.91 V and 14.8 mA/cm^2^ of the same c-Si PV cell combined with pure PMMA and CuInS_2_/ZnS QDs-LSC respectively. More importantly, the PCE of LSC-PV device has been increased from 2.73% for pure PMMA to 8.71% for CuInS_2_/ZnS QDs-LSC as much as more than 3 folds. The performance enhancement is mainly due to the addition of CuInS_2_/ZnS QDs-LSC which absorbs light with short wavelength and emits light with long wavelength that is more sensitive for the c-Si PV cell. Moreover, though the PCE value of the new CuInS_2_/ZnS QDs-LSC-PV device (8.71%) is lower than that of commercial PV cell (13%), the solar harvest area is enlarged for 7.33 folds to the same size c-Si PV cell, which means we can harvest 4.91 folds solar energy with the assistance of the CuInS_2_/ZnS QDs-LSC for the same size c-Si PV cell. In other words, only much smaller size c-Si PV cell is needed by using CuInS_2_/ZnS QDs-LSC to generate the same electrical power, which will reduce the cost of solar photovoltaic system dramatically.

## Conclusions

In this research, a new type of QDs-LSC integrated with heavy metal free CuInS_2_/ZnS core/shell QDs with large Stokes shift (larger than 150 nm) and high photoluminescence quantum yield (81%) has been proposed for the first time. Performance both of CuInS_2_/ZnS QDs and its related QDs-LSC are described and analyzed in detail. The optical efficiency of the new CuInS_2_/ZnS QDs-LSC reaches as high as 26.5%. Moreover, the power conversion efficiency of the c-Si PV cell attached at the side of the LSC increases as much as more than 3 folds from 2.73% for pure PMMA-PV device to 8.71% for the CuInS_2_/ZnS QDs-LSC-PV device. Furthermore, the PV device is able to harvest 4.91 folds solar energy with the assistance of the new CuInS_2_/ZnS QDs-LSC for the same size c-Si PV cell. This new CuInS_2_/ZnS QDs-LSC provides an effective way for the high efficiency, nonhazardous and low cost solar energy.

## Methods

### Raw materials

Copper (Ι) iodide (CuI, 99.999%), indium (ΙΙΙ) acetate (In(OAc)_3_, 99.99%), zinc stearate (Zn(St)_2_, 10–12% Zn basis), 1-dodecanethiol (DDT, 98%), methyl methacrylate (MMA, 99%), 2, 2′-azobis(2-methylpropionitrile) (AIBN, 99%), paraffin liquid, *n*-hexane, chloroform and absolute ethanol were used as raw materials. All chemicals were used as received without any further purification.

### Synthesis of core/shell CuInS_2_/ZnS QDs

The CuInS_2_/ZnS QDs were synthesized by first preparing the CuInS_2_ core and then carried out a slow high-temperature ZnS shell growth.

All synthesizes were performed in the non-coordinating solvent paraffin liquid under an argon atmosphere using the standard Schlenk techniques. CuI (0.25 mmol), In(OAc)_3_ (1 mmol), DDT (10 mL) were mixed with paraffin liquid (10 mL) in a 50 mL three-neck flask and then degassed at 120 °C for 30 min and then Ar-purged. The mixture was then heated to 250 °C for 3 min. With the temperature increased, the color of the reaction solution gradually changed from slight green to transparent orange, red and finally brownish red, and the CuInS_2_ core were synthesized.

Subsequently, for the synthesis of CuInS_2_/ZnS core/shell QDs, Zn(St)_2_ (16 mmol), DDT (8 mL) and paraffin liquid (16 mL) were mixed and added to the core solution under argon atmosphere, and then the mixture was heated to 260 °C and maintained for 120 min to obtain final core-shell QDs. The QDs were purified and stored for further use.

### Fabrication of CuInS_2_/ZnS QDs-PMMA bulk composites

A practical device of efficient CuInS_2_/ZnS-LSC requires the incorporation of QDs into high optical quality transparent matrix, such as polymethyl methacrylate (PMMA). The optical-grade PMMA is typically produced by the bulk polymerization of MMA in the presence of thermal radical initiators, such as az-compounds and peroxides, which carried out in a thermostatic water bath. To generate homogeneous and transparent nanocomposites, it is necessary to transfer the nanocrystals into the monomer solution to form to a stable and homogeneous dispersion before the process of polymerization. For thermal polymerization of QD-PMMA, the process was characterized by two steps, called pre-polymerization and post-polymerization.

Firstly, MMA (20 mL) monomer and AIBN (0.2% wt/wt with respect to MMA) were added to a 50 mL beaker and kept stirring until the AIBN was dissolved completely. Then the mixture was transferred into a 50 mL three-neck round-bottom flask. Subsequently, CuInS_2_/ZnS QDs chloroform solution was dropwise added into the flask and the mixture was homogeneously dispersed by the ultrasound treatment. After that, the flask was placed into the thermostatic water bath at 70 °C for the desired reaction time and cooled to room temperature when the mixture reached certain viscosity. Then, the viscous liquid was introduced into the casting mould. Secondly, the casting mould was placed in the vacuum oven at 45 °C and kept at this condition for 24 h. Finally, the nanocomposite was heated to 70 °C overnight. Then the resulting composite was cut into squares and polished for optical measurement so that LSC device was obtained.

### QDs-LSC-PV device

The PMMA-QDs composite was obtained, tailored and polished to 22 mm × 22 mm × 3 mm bulk sharp to achieve QDs-LSC. The sunlight receiving panel of c-Si solar cell was pasted on one of 22 mm × 3 mm faces. One 22 mm ×22 mm face of QDs-LSC was exposured under the solar simulate light source for further tests.

### Characterization

The High Resolution Transmission Electron Microscopy (HRTEM) was carried out on a JEOL JEM-2100 F (Cs) microscope operating at 200 kV. The ultraviolet-visible (UV-Vis) absorption spectra were measured using a TU-1901 UV-Vis spec-trophotometer over the scan range 250–800 nm and a resolution of 1.0 nm. The excitation and emission spectra were carried out using a FluoroSENS-9000 photoluminescence spectrophotometer with a static xenon lamp (150 W) as an excitation source. The PL QY of QDs was performed using a quantum yield measurement system (FluoroSENS-9000 photoluminescence spectrophotometer) with a 150 W xenon lamp coupled to a monochromator for wavelength discrimination, a 120 mm integrating sphere as sample chamber and a multichannel analyzer for signal detection. Solar simulator with an air mass 1.5 global illumination (AM 1.5 G, 100 mW/cm^2^) and a Keithley 2400 source meter were used for the J–V characterization. All the measurements were carried out at room temperature.

## Additional Information

**How to cite this article**: Li, C. *et al*. Large Stokes Shift and High Efficiency Luminescent Solar Concentrator Incorporated with CuInS_2_/ZnS Quantum Dots. *Sci. Rep*. **5**, 17777; doi: 10.1038/srep17777 (2015).

## Figures and Tables

**Figure 1:**
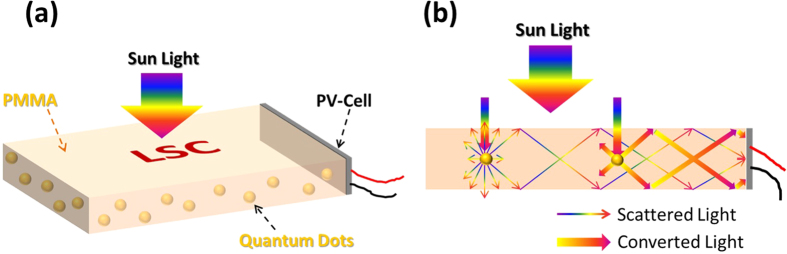
(**a**) Schematic of the conventional flat QDs-LSC-PV device and illustrates the principle of the device. (**b**) Three behaviors of light in this device: light converting, Rayleigh scattering and light guiding.

**Table 1:** Comparison of performance between QDs-LSC-PV device and pure PMMA-PV device.

TYPES	*V*_OC_(V)	*J*_SC_(mA/cm^2^)	FF (%)	PCE (%)
*QDs-LSC-PV*	0.91	14.8	64.7	**8.71**
*pure PMMA-PV*	0.72	7.2	52.6	2.73
